# The Global Diet Quality Score predicts diet quality of women of reproductive age in Addis Ababa, Ethiopia

**DOI:** 10.1017/S0007114523000508

**Published:** 2023-11-14

**Authors:** Kaleab Baye, Zemenu Yaregal

**Affiliations:** 1 College of Natural and Computational Sciences, Center for Food Science and Nutrition, Addis Ababa University, Addis Ababa, Ethiopia; 2 Department of Food Science and Applied Nutrition, Addis Ababa Science and Technology University, P O, Box 16417, Addis Ababa, Ethiopia

**Keywords:** Diet quality, Unhealthy diet, Healthy diet, Ultra-processed foods, Low income, Nutrient adequacy, Non-communicable diseases

## Abstract

Improving diet quality is recognised as a double-duty action that can simultaneously address multiple forms of malnutrition. This study aimed to assess diet quality among non-pregnant non-lactating women of reproductive age (WRA) in Addis Ababa, Ethiopia. A 1-d quantitative 24 h recall was conducted among 653 non-pregnant/non-lactating women. Diet quality, assessed using the women dietary diversity score (WDDS), the Global Diet Quality Score (GDQS) and the Nova 4 classification reflecting consumption of ultra-processed foods (UPF), was compared. The proportion that meets the minimum dietary diversity for women (MDD-W) was estimated. The average MDD-W score was 2·6 (sd 0·9), with only 3 % of women meeting the MDD-W (≥ 5 food groups). Consumption of wholegrain and legumes was high, but UPF were also consumed by 9 % of the women. GDQS was positively associated with WDDS, age and skipping breakfast and was negatively associated with eating out of home and UPF consumption (*P* < 0·05). The multivariate regression model showed that GDQS (total) was not associated with wealth but was significantly associated with both UPF and WDDS (*P* < 0·001). Unlike UPF and WDDS alone, GDQS was able to predict both nutrient adequacy and unhealthy dietary practices. The diet quality of WRA in Addis Ababa is low in diversity, possibly exposing them to higher risk of nutrient inadequacy and non-communicable diseases as reflected by the low GDQS. Understanding what drives food and dietary choices in urban settings is urgently needed.

Poor quality diets are increasingly recognised as the major drivers of the global burden of morbidity and mortality^(1,2)^. In 2017, 22 % of the age-standardised adult mortality was attributable to dietary risks, making poor diets the highest risk factor to adult mortality^(3)^. Poor diets have been linked to nutrient inadequacy, overweight/obesity and non-communicable diseases (NCD)^(4)^. Poor diets are thus considered a shared driver for all forms of malnutrition^(5)^. Consequently, improving diets is increasingly recognised as a double-duty action, required to simultaneously address multiple forms of malnutrition^(4)^.

Despite the need for a good diagnostics of current dietary practices, the high expense associated with implementing dietary intake assessments has made such data scarce. Considering that what is not measured cannot be improved, easy-to-use, low-burden diet quality indicators are needed. Efforts towards this end led to the development of various indictors over the past decade. For example, the minimum dietary diversity for women (MDD-W) was developed to assess the nutrient adequacy of women’s diet based on a women dietary diversity score (WDDS)^(6)^. However, this indicator was not developed to capture unhealthy practices, and hence, it is not suitable for predicting risk of overweight/obesity and NCD^(7)^. On the other hand, indicators like the NOVA classification, particularly NOVA 4 groups (ultra-processed food (UPF)), were found to be associated with overweight/obesity and NCDs, but their association with nutrient adequacy is less clear^(8)^.

More recently, the Global Diet Quality Score (GDQS) was developed to fill this critical gap of a universal, simple-to-use, indicator predicting both nutrient adequacy and risk of NCD^(9)^. The GDQS is composed of twenty-five food groups with a more specific distinction between healthy and unhealthy foods and awards points for healthy foods while subtracting points for harmful foods. The GDQS, unlike the earlier indicators, also takes into account amounts consumed^(10)^. The GDQS was recently validated with data from ten Sub-Saharan African countries, but this relied on estimates from a FFQ and was limited to rural residents that are less exposed to unhealthy foods^(11)^. More recently, the GDQS’s ability to predict nutrient adequacy was assessed in Ethiopia, and the findings suggested that the GDQS was associated with energy-adjusted nutrient intakes, but the authors called for more studies^(10)^. Given that this study aimed to validate GDQS, it did not provide a description of the diet quality of women in Ethiopia and conflated urban and rural with more samples from rural areas. Given the rapid rise in overweight/obesity in Addis Ababa, the association between GDQS and other diet quality metrics including the NOVA classification is needed in this setting.

The objectives of the study were two-folds and aimed:

- to characterise the consumption of women of reproductive age living in Addis Ababa, using three diet quality metrics and indicators: (i) WDDS, (ii) UPF (NOVA classification) and (iii) the GDQS.
- to compare how the recently developed GDQS compare to existing diet quality metrics like WDDS and NOVA-4 (UPF), socio-economic factors, and dietary practices like snacking, and eating out of home.

Consequently, this study provides much needed information on the diet quality of women in Addis Ababa, using diet quality metrics/indicators aimed to assess nutrient inadequacy (WDDS/MDD-W), unhealthy dietary practices (UPF), and overall diet quality (GDQS). Besides, this study illustrated how the various diet quality metrics compare to each other, and further explores factors associated with GDQS.

## Methods

### Subjects and study design

A population-based cross-sectional food consumption survey was conducted in Addis Ababa, the capital city of Ethiopia, in September–October 2019. A multi-stage sampling procedure was used to select study participants. The sampling process began with the selection of sub-cities using random sampling. A systematic random sample was applied to choose the households in the second stage, and one eligible subject was randomly chosen from each household. This study was part of a wider study that included a total of 996 adults from 1060 households. The 653 women were selected from the full list if they met the following criteria: i) aged 15–49 years and ii) non-pregnant/non-lactating. Pregnant and lactating women were excluded because the diet quality metrics used in this study (GDQS/MDD-W) were developed for non-pregnant/non-lactating women.

### Socio-demographic characteristics

Information on socio-demographic characteristics was collected through an interviewer-administered questionnaire. General information about the age, sex, employment, and house ownership and education status was collected. The questionnaires were pretested and translated to *Amharic* language.

### Food consumption survey

Type of foods and beverages and the amounts consumed during the previous day were obtained using an interactive, quantitative 24-h dietary recall, which applied the multiple-pass technique following the method described in Gibson and Ferguson^(12)^. On the first pass, participants were asked to list all the foods consumed in the prior day. The second pass, described the ingredients and processing of the foods, followed by portion size estimation in the third pass. Portion size was estimated, whenever possible, using salted replicas of actual foods weighed in the households using kitchen scales (Kinlee ACS-EK01). Otherwise, local utensils and graduated models were used. Using standard recipes, portion sizes were estimated using local household utensils, cups, dry foods (e.g. rice) and food photographs. In the final (fourth) pass, completeness of the collected information was checked.

The prevalence of snaking, defined as meals eaten in addition to major meals (breakfast, lunch and dinner), and the practice of eating out of home were assessed.

### Diet quality assessment

#### Women dietary diversity score and minimum dietary diversity for women

The foods consumed were categorised into one of the following ten food groups: (1) grains, white roots and tubers, and plantains; (2) pulses (beans, peas and lentils); (3) nuts and seeds; (4) milk and milk products; (5) meat, poultry and fish; (6) eggs; (7) dark green leafy vegetables; (8) other vitamin A-rich fruits and vegetables; (9) other vegetables and (10) other fruits (FAO 2021). The proportion of women who met the MDD-W, that is, consuming at least five out of the ten food groups, was estimated. A food was counted into the food group if more than 15 g was consumed.

#### The Global Diet Quality Score

The GDQS was recently developed to be sensitive to diet-related outcomes associated with all forms of malnutrition. The GDQS was recently shown to capture both nutrient adequacy and diet-related NCD risks^(13)^. To construct the GDQS, we classified foods consumed in the previous 24-h recall into twenty-five food groups: sixteen healthy food groups (GDQS+), seven unhealthy food groups (GDQS-) and two food groups (red meat, high-fat dairy) that are unhealthy when consumed in excessive amounts^(14)^. To this end, we used the GDQS: data collection options and tabulation guidelines to assess the risk of nutritional inadequacy and NCD-related outcomes^(10,14)^.

The mean values and standard deviations for the overall GDQS as well as the two GDQS sub-metrics (GDQS+ and GDQS–) were determined. The total GDQS was computed by adding the points from each of the twenty-five GDQS food groups, with a range of 0–49. The overall score across the sixteen healthy GDQS+ food groups, with a possible range of 0–32, was used to determine the GDQS+. With a possible range of 0–17, the GDQS- was determined from the overall score across the seven unhealthy food categories and the two food groups that are unhealthy when taken in excess amounts.

Finally, population-based cutoffs of 15 and 23 were applied to allow for reporting of the percentage of the population at risk for nutrient inadequacy and NCD-related outcomes. According to the guideline, a GDQS ≥ 23 is associated with a low risk of nutrient inadequacy and NCD-related outcomes, scores ≥ 15 and < 23 indicate moderate risk and scores < 15 indicate high risk^(9)^. For each of the GDQS food groups, the percentage of people who consumed low, moderate and high (and very high, in the case of high-fat dairy) amount was also provided. For all food groups that were not reported by a responder, a value of zero grams was assigned to the quantity consumed, with the exception of liquid oil. Liquid oil data were gathered directly from respondent reports and based on the guidelines’ algorithm^(10)^.

#### NOVA classification: Ultra-processed foods

Food and beverages consumed in the last 24 h were categorised into the NOVA 4 classification^(8)^, following the standard definition: ‘foods that are formulations of ingredients, mostly of exclusive industrial use, that result from a series of industrial processes’. Given recent evidences suggesting that the most prominent adverse health effects are related to the consumption of foods categorised under NOVA 4 (UPF)^(15)^, we estimated the prevalence of UPF consumption and explored its association with the GDQS. The foods that constituted the UPF category are presented in online Supplementary Fig. S1.

### Ethics

This study was conducted according to the guidelines laid out in the Declaration of Helsinki, and all procedures involving human subjects or patients were approved by the International Food Policy Research Institute (IFPRI) institutional ethics review committee (ref # MTID-20-0413). Written informed consent was obtained from all subjects/patients after explaining the purpose and methods of the study.

### Quality control and statistical analyses

The food consumption survey was conducted by an experienced survey team that received extensive training, followed by a pretest. The questionnaires were translated into *Amharic*, completeness of the data collected was checked daily and data were double-entered and cleaned. Descriptive statistics of continuous and categorical variables were presented as mean/median or in frequency/percentages, respectively. The normality of the distribution of the data was checked. Multicollinearity tests were run. The variance inflation factor was all below 0·2, suggesting that multicollinearity was not an issue. Associations between diet quality indicators and other variables were run using Pearson or spearman correlations, followed by a multi-linear regression analyses. All the covariates included in the Pearson/spearman correlation were based on prior evidence of their relationship with diet quality. All the covariates had a *P*-value of < 0·2 in the bivariate model and thus were also included in the multi-linear regression model. Data were analysed using excel and SPSS version 23. *P*-values < 0·05 were considered statistically significant.

## Results

The basic characteristics of the women of reproductive age (15–49 years) who participated in the study are presented in Table 1. The mean age of the women was 32·7 (sd 7·8) years, with more than two-third of the participants being in the 20–40 years age range. More than two-thirds had secondary or higher education level. Close to half of the participants reported to have owned the house they live-in, whereas over half lived in rented houses.

**Table 1.** Characteristics of study participants (*n* 653) (Numbers and percentages)

Variable	*n*	%
Age (years)		
15–19	40	6
20–29	193	30
30–39	264	40
40–49	156	24
Education level		
No education	74	11·6
Primary	197	30·6
Secondary/higher	373	57·8
Main occupation		
Employee (public/private/NGO)	184	28·6
Self-employed (Own business)	102	15·8
Unpaid housework	176	27·3
Student	72	11·2
Other	110	17·1
House ownership		
Own house	301	46
Rented house	104	16
Government rent	230	35
Live with family	18	3
Home gardening*		
Yes	77	12
No	576	88
Sub-city		
Addis ketema	98	15
Akaki	106	16
Bole	159	25
Kirkos	89	14
Ledeta	57	9
Yeka	144	22

* Growing vegetables in their home gardens.

Table 2 presents the dietary characteristics of the women of reproductive age. About half consumed snacks and about a third consumed at least one meal outside home. The dietary diversity was very low as the mean dietary diversity was 2·6, and only 2·5 % of our subjects met the MDD-W. The median (Q1, Q3) GDQS score was 17 (15, 19). Close to a third (27 %) had a GDQS score suggestive of a high risk of poor dietary outcomes. Very few (3 %) had a GDQS suggestive of a low risk for poor dietary outcome.

**Table 2.** Dietary characteristics of women of reproductive age in Addis Ababa, Ethiopia (Numbers and percentages; mean values and standard deviations)

Dietary patterns	*n*	%
Snacking	321	49
Out of home consumption	177	27
UPF	57	9
WDDS		
Mean	2·6	
sd	0·9	
MDD	16	2·5
GDQS		
Median	17·5	
Q1, Q3	15, 19	
GDQS+		
Median	6·0	
Q1, Q3	4·5, 8·0	
GDQS		
Median	11	
Q1, Q3	10,12	
GDQS < 15 (high risk)	175	27
GDQS 15–22	459	70
GDQS > 23 (low risk)	19	3

MDD, minimum dietary diversity for women; GDQS, Global Diet Quality Score (out of a maximum score of 32); UPF, ultra-processed foods; WDDS, women dietary diversity score (out of ten food groups).

Snacking is defined as taking food between major meals like breakfast, lunch and dinner.

Risk of poor dietary outcome.

**Fig. 1.**
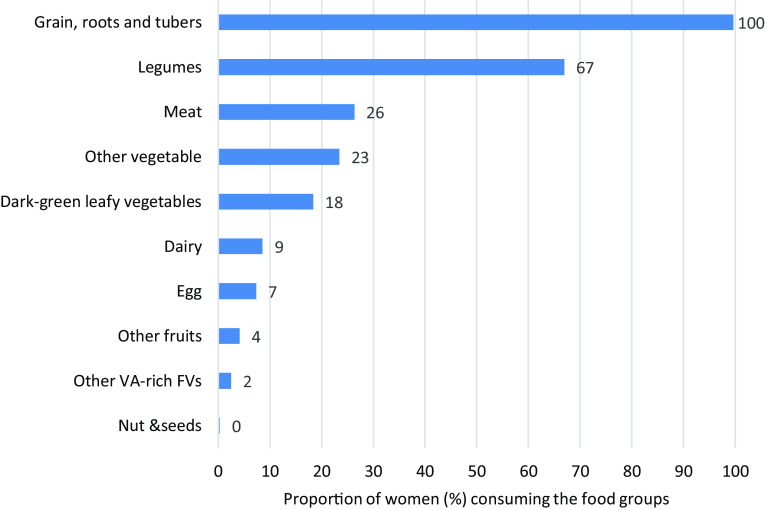
Food groups consumed by women of reproductive age in Addis Ababa, Ethiopia. VA, vitamin A-rich; FV, fruit and vegetables.

Table 3 presents the proportion of the population consuming low, medium and high (very high) quantities of healthy (GDQS+) and unhealthy (GDQS-) foods. Consumption of fruits, vegetables, fish, poultry and eggs was very low. This was in line with the findings from the food group distribution using the ten food groups of the MDD-W (Fig. 1). In contrast, whole grain and legume consumption was high. Regarding foods considered unhealthy (in excess amounts), high-fat dairy and meat consumption was low, whereas refined grains, baked goods and sweets were consumed in high quantities by close to half of the population.

**Table 3.** Distribution of the categories for Global Diet Quality Score (*n* 653) (Numbers and percentages)

Inclusion in metrics	Scoring classification	Food group	*n*	%	% of the population consuming low, medium, high (very high) quantities			
					Low	Middle	High	Very high
GDQS+	Healthy	Citrus fruits	6	1	99	0·6	0·3	NA
		Deep orange fruits	1	0·2	99·8	NA	0·2	NA
		Other fruits	22	3	97	1·2	2	NA
		Dark green leafy vegetables	111	18	83	6	11	NA
		Cruciferous vegetables	32	5	95	1	4	NA
		Deep orange vegetables	46	7	93	2·5	4·5	NA
		Other vegetables	124	19	81	6	13	NA
		Legumes	445	68	32	4	64	NA
		Deep orange tubers	19	3	97	1	2	NA
		Nuts and seeds	2	0·3	99·7	0·3	NA	NA
		Whole grains	651	99·5*	0·3	0·2	99·5	NA
		Liquid oils	191	29	71	1	28	NA
		Fish and shellfish	2	0·3	99·8	0·1	0·1	NA
		Poultry and game meat	13	2	98	NA	2	NA
		Low-fat dairy	17	3	97·5	1	1·5	NA
		Eggs	49	7·5	92·5	1·5	6	NA
GDQS-	Unhealthy in excess amounts	High-fat dairy	60	9	91	5	4	0·1
		Red meat	5	1·5	98·5	1	0·5	NA
	Unhealthy	Processed meat	154	24	76	NA	24	NA
		Refined grains and baked goods	312	48	52	3	45	NA
		Sweets and ice cream	549	84	16	31	53	NA
		Sugar-sweetened beverages	11	2	98	NA	2	NA
		Juice	2	0·3	99·6	0·3	NA	NA
		White roots and tubers	185	28	72	12	16	NA
		Purchased deep fried foods	12	2	98	1·1	1	NA

* Consumption of injera (staple) prepared from fermented wholegrain teff flour.

The association between selected socio-demographic characteristics, dietary practices and GDQS is presented in Table 4. Age, WDDS, skipping breakfast and eating meals out of home were significantly associated with GDQS total score, but the strength of the associations was (very) weak. The strength of the association between WDDS and GDDQ+ was stronger (*r* = 0·53) than the one with the total GDQS score (*r* = 0·34). Consumption of UPF was inversely associated with GDQS. Wealth score was, however, not associated with any of the GDQS scores but was weakly associated with MDD-W (online Supplementary Table S1). Table 5 presents the multiple linear regression model exploring the association of various factors with GDQS. The association between GDQS and WDDS, snacking, skipping breakfast, eating-out and UPF remained significant (*P* < 0·05). The association between GDQS+ and WDDS, as well as that of GDQS- and UPF remained significant and strong (*P* < 0·001).

**Table 4.** Association between GDQS and dietary and socio-demographic characteristics (95 % confidence intervals)

Variables	GDQS total			GDQS+			GDQS-		
	*r*	95 % CI	*P*	*r*	95 % CI	*P*	*r*	95 % CI	*P*
Age	0·14	0·06, 0·21	<0·001	0·1	0·03, 0·18	0·009	0·11	0·03, 0·18	0·006
Wealth score	–0·02	–0·09, 0·06	0·598	–0·01	–0·08, 0·07	0·860	–0·03	–0·11, 0·05	0·465
WDDS	0·34	0·27, 0·41	<0·001	0·53	0·47, 0·58	<0·001	–0·05	–0·13, 0·02	0·168
Snacking	–0·05	–0·13, 0·03	0·199	–0·03	–0·1, 0·05	0·476	–0·05	–0·13, 0·02	0·17
Skipping breakfast	0·11	0·04, 0·19	0·004	0·00	–0·08, 0·08	0·995	0·21	0·13, 0·28	<0·001
Eating out	–0·13	–0·21, –0·06	<0·001	–0·09	–0·17, –0·01	0·02	–0·12	–0·19, –0·04	0·003
UPF	–0·27	–0·34, –0·2	<0·001	–0·13	–0·21, –0·06	<0·001	–0·31	–0·38, –0·24	<0·001

GDQS, Global Dietary Quality Score (+: healthy; -: unhealthy); UPF, ultra-processed foods; WDDS, women dietary diversity score.

**Table 5.** Association between dietary and socio-demographic characteristics with GDQS, multiple linear regression (95 % confidence intervals)

Variables	GDQS total			GDQS+			GDQS-		
	*β*	95 % CI	*P*	*β*	95 % CI	*P*	*β*	95 % CI	*P*
Age	0·04	0·01, 0·06	0·009	0·02	0·00, 0·04	0·036	0·02	–0·00, 0·03	0·072
Wealth									
Poorest (Ref.)									
Poorer	0·47	–0·18, 1·11	0·154	0·11	–0·36, 0·59	0·641	0·35	–0·03, 0·74	0·073
Middle	0·29	–0·36, 0·94	0·386	0·16	–0·31, 0·64	0·499	0·12	–0·27, 0·52	0·54
Richer	–0·28	–0·97, 0·41	0·42	–0·42	–0·92, 0·07	0·092	0·14	–0·27, 0·55	0·504
Richest	–0·33	–1·01, 0·36	0·35	–0·54	–1·06, –0·02	0·041	0·21	–0·20, 0·63	0·312
WDDS	1·54	1·26, 1·82	<0·001	1·58	1·34, 1·81	<0·001	–0·04	–0·19, 0·12	0·647
Snacking	–0·31	–0·76, 0·14	0·182	–0·11	–0·44, 0·23	0·532	–0·2	–0·46, 0·06	0·131
Skipping breakfast	1·01	0·53, 1·49	<0·001	0·25	–0·12, 0·61	0·184	0·76	0·49, 1·04	<0·001
Eating out	–1·25	–1·75, –0·74	<0·001	–0·94	–1·31, –0·56	<0·001	–0·31	–0·61, –0·01	0·042
UPF	–2·94	–3·85, –2·02	<0·001	–1·15	–1·82, –0·49	0·001	–1·78	–2·26, –1·31	<0·001
Constant	12·28	11·09, 13·48	<0·001	1·93	1·01, 2·86	<0·001	10·35	9·66, 11·04	<0·001

WDDS, women dietary diversity score (calculated out of ten food groups); UPF, ultra-processed foods.

## Discussion

The present study has characterised the dietary quality of women of reproductive age residing in the capital city, Addis Ababa, Ethiopia. The dietary diversity of the women was very low, suggesting potentially high nutrient inadequacy. Besides, the wide consumption of refined cereals and sugary foods, along with the poor diversity, is likely to predispose women not only to a serious risk of inadequate nutrient intake but also to NCD. On the other hand, consumption of wholegrain and legumes was high. GDQS was positively associated with WDDS, age and skipping breakfast, whereas it was negatively associated with eating out of home and UPF. The GDQS was, however, not associated with wealth.

Despite reductions in undernutrition, Ethiopia has been witnessing a rapid rise in overweight/obesity that is disproportionately affecting women living in urban areas^(16)^. Addis Ababa has seen the fastest rise in overweight/obesity over the last decade, sounding the alarm of a possible nutrition transition in action. Earlier surveys have identified that consumption of fruits and vegetables was extremely low, but more thorough dietary assessments were limited^(17)^. Using the MDD-W, a validated indicator for nutrient inadequacy, our study has confirmed the low dietary diversity in urban areas, despite better knowledge and improved market access^(18)^. Such low diversity can not only increase nutrient deficiencies, but can, through low fruit and vegetable consumption, also increase the risk of NCD. Unfortunately, the MDD-W is ill-equipped to assess the later, but recent development of the GDQS indicator capturing both healthy and unhealthy food consumption can fill this gap^(10)^.

The stronger association between WDDS and GDQS+ compared with the overall score, and the null association with GDQS- not only confirms the WDDS ability to predict the consumption of healthy food but also illustrates that WDDS poorly performs in predicting unhealthy food consumption^(9)^. Our study highlighted that close to a third of our sample had GDQS scores < 15, suggesting a high risk of poor dietary outcomes, hence indicating an increased risk for both forms of malnutrition. Surprisingly, only 3 % of the women in our study had a diet with low heath risk (GDQS > 23). This is unfortunate and indicates the urgent need for interventions that aim to improve urban diets.

Interestingly, women’s age, snacking and eating out of home were weakly associated with GDQS, but GDQS was not associated with wealth. This may suggest that a poor diet in this urban area is not necessarily the outcome of unaffordability of healthy foods, or perhaps a healthy diet is unaffordable for a large share of the urban residents. Indeed, a recent study that assessed the affordability of an optimised minimum-cost nutritious diet showed that 60–65 % of residents in Addis Ababa could not afford the minimum-cost nutritious diet^(19)^. Considering that healthier diet is a step further in the diet quality ladder, it can be assumed that a larger share of Addis Ababa residents may not afford a healthy diet. In addition, the food environment, convenience, taste preference and knowledge of consumers can affect food choices and, hence, the healthiness of the diet. This calls for a more comprehensive assessment of the food system and influencers of food choice to inform the design of more effective urban-focused nutrition interventions that address all forms of malnutrition.

The present study has a number of strengths and limitations that need to be considered when interpreting our findings. First, the cross-sectional nature of our study does not allow causal inferences to be made. Second, although we conducted a quantitative 24 h recall in a relatively large sample of women in Addis Ababa, we had only a 1-d 24 h recall, which does not allow us to determine the usual intake. Third, although the association of the GDQS with nutrient intakes could have been further explored, our study focused on food-based indicators. The strengths of our study include the large sample size and the application of quantitative 24 h recall. In addition, the assessment of the diet quality with MDD-W, UPF (nova classification) and the recently validated GDQS in an African urban setting makes our study unique. Our study can serve as a baseline for the monitoring diets in Addis Ababa.

Our study fills a critical knowledge gap regarding the diet quality of women in urban settings and highlighted that diet quality of women in Addis Ababa is low, possibly exposing women in Addis Ababa to higher risk of nutrient inadequacy and NCD. Understanding what drives food and dietary choices in urban settings is urgently needed to effectively implement interventions that improve diet quality not only in Addis Ababa but also in other urban areas of Ethiopia. The GDQS can allow a more effective monitoring of dietary changes and better tracking of upcoming food systems’ related interventions.

## Supplementary material

For supplementary material accompanying this paper visit https://doi.org/10.1017/S0007114523000508. click here to view supplementary material
