# Supplementary material for: The Global Diet Quality Score predicts diet quality of women of reproductive age in Addis Ababa, Ethiopia
Source: Br J Nutr. 2023 Mar 6;130(9):1573–9. doi: 10.1017/S0007114523000508 (PMC10551468; doi:10.1017/S0007114523000508)
Supplement: Supplementary file 1 [file S0007114523000508sup001.docx]

**Supplementary tables and figures**

Table S1 Mean diffrences of GDQS across components

| **Variables** | | **GDQS total** | | **GDQS+** | | | **GDQS-** | | |
| --- | --- | --- | --- | --- | --- | --- | --- | --- | --- |
|  |  | **mean [95%CI]** | **P-value** | **mean [95%CI]** | **P-value** | **mean [95%CI]** | | **P-value** |  |
| Snacking | no | 17.2 [16.9, 17.5] | 0.199 | 6.3 [6, 6.6] | 0.476 | 10.9 [10.7, 11.1] | | 0.17 |  |
|  | yes | 16.9 [16.5, 17.2] |  | 6.1 [5.9, 6.4] |  | 10.7 [10.5, 10.9] | |  |  |
| Skipping breakfast | no | 16.8 [16.5, 17.1] | 0.004 | 6.2 [6, 6.4] | 0.955 | 10.6 [10.4, 10.7] | | <0.001 |  |
|  | yes | 17.6 [17.1, 18] |  | 6.2 [5.9, 6.6] |  | 11.4 [11.1, 11.6] | |  |  |
| Eating out | no | 17.3 [17, 17.6] | <0.001 | 6.4 [6.1, 6.6] | 0.02 | 10.9 [10.8, 11.1] | | 0.003 |  |
|  | yes | 16.3 [15.8, 16.8] |  | 5.8 [5.5, 6.2] |  | 10.5 [10.2, 10.8] | |  |  |
| UPF | No | 17.3[17.1, 17.6] | <0.001 | 6.3[6.1, 6.5] | <0.001 | 11[10.9, 11.1] | | <0.001 |  |
|  | Yes | 14.2[13.2, 15.1] |  | 5.1[4.4, 5.8] |  | 9.1[8.6, 9.5] | |  |  |

P-values are from independent student t-test

Correlation between wealth score and MDDW

| Variable | **MDDW** | |
| --- | --- | --- |
|  | r [95%CI] | P-value |
| Wealth score (0-10)* | 0.21[0.14, 0.28] | <0.001 |
| **Spearman's  correlation coefficients* | | |

**Figure S1** Type and share (%) of ultra-processed foods consumed
